# Psychological Capital Mediates the Association between Job Stress and Burnout of among Korean Psychiatric Nurses

**DOI:** 10.3390/healthcare8030199

**Published:** 2020-07-06

**Authors:** Sooyeong Kim, YoungRan Kweon

**Affiliations:** Department of Nursing, Chonnam National University, Gwangju 61469, Korea; tndud914269@hanmail.net

**Keywords:** psychiatric nurse, job stress, psychological capital, burnout, bootstrapping, mediating effect

## Abstract

This study examined the mediating effect of psychological capital in the relationship between job stress and burnout of psychiatric nurses. The participants were 108 psychiatric nurses working in three psychiatric hospitals located in South Korea. Data were collected from 10 August to 15 September 2018 using self-report questionnaires. Data were analyzed using descriptive statistics, *t*-test, one-way ANOVA, Pearson’s correlation coefficient, and multiple linear regression by IBM SPSS 24.0 program. In addition, a bootstrapping test using the SPSS PROCESS macro was conducted to test the statistical significance of the mediating effect. There was significant correlation between job stress, psychological capital, and burnout. Psychological capital showed partial mediating effects in the relationship between job stress and burnout. Job stress explained 29.7% of the variance in burnout, and the model including job stress and psychological capital explained 49.6% of the variance in burnout. The bootstrapping showed that psychological capital was a significant sub-parameter and decreased job stress and burnout (LLCI = −0.1442, ULCI = −0.3548). These findings suggest that psychiatric nurses’ burnout can be reduced by implementing various health care programs designed to increase psychological capital.

## 1. Introduction

Due to the rapid social change, people are experiencing stress, conflict and many other mental health problems. Mental health has recently emerged as a social problem as these phenomena accumulate, and various policies at the national level have been proposed to solve it. Additionally, the production of highly qualified professionals is one of the prerequisites for the implementation of such national health-related policies. The change in societal attitude towards mental health issues has led to an increase in expectations and the duties for mental health nurses [1]. Consequently, demand for quality mental health care has become a new stressor for nurses.

Nursing has been known as one of the most stressful professions. In particular, psychiatric nurses who use themselves as therapeutic tools have higher job stress compared to nurses from other specialties. The nurses in the psychiatric unit experience high levels of job stress as compared to nurses in other wards [2,3]. Mental health nurses also experience more tension and increased job demands than nurses in general wards, as their patients encounter frequent relapses, chronicizations, and repeated hospitalizations. Additionally, psychiatric nurses often experience helplessness and are trained to prepare for psychiatric emergencies, such as patients with a tendency of harming themselves and others [4].

Psychiatric nurses are responsible for taking actions in order to restore patients’ mental equilibrium when it comes to self-harm and misbehavior. Nonetheless, experiencing stress associated with potential violence or an unsafe work environment for psychiatric nurses is inevitable [5]. Excessive stress can lead to burnout, and this can result in either them leaving their jobs or retirement. A psychiatric nurse is exposed to experiencing high levels of burnout. Burnout in a health care setting not only leads to reduced effectiveness at work, but may also restrict the perception of the individual, affecting a person’s judgment, and decreasing the quality of care [6]. Therefore, efforts to improve the working environment for mental health nurses and to change the internal factors of the nurses themselves are imperative.

Recently, research on psychological capital has been conducted in an effort to reduce the burnout of nurses [7]. Psychological capital can be seen as an important human resource. Psychological capital is a positive psychological state that utilizes the psychological strengths of an individual to achieve goals and drive performance. The constituent of psychological capital is the potential for self-development with self-efficacy, optimism, hope and resilience. It is a complex psychological capacity that positively affects individuals and organizations through positive cognition [8]. This has a positive effect on nurses who are physically and mentally weak in clinical practice and experience physical burnout and stress [9]. The constituents of psychological capital can give positive meaning to psychiatric nurses who feel sadness and despair while watching patients relapse and experience chronicization frequently. Psychological capital has also been found to be linked to job stress, burnout and intention to resign among nurses [7,8,9,10].

Currently, few studies have been undertaken on the relationship between psychological capital and job burnout. In addition, the body of research concerning the relationship between psychological capital and burnout remains relatively small. However, only a partial analysis of the relationship between psychological capital and variables, such as job stress and burnout, has been performed [7,11,12]. In addition, there was no concrete verification of the effect of psychological capital on the relationship between job stress and burnout. In order to reduce the burnout of nurses, it is necessary to confirm that psychological capital is an important parameter based on previous findings that internal factors are more important than external factors [13]. Factors related to burnout, such as personal characteristics, demographic characteristics, health characteristics, and motivations among nurses are difficult to change. However, psychological capital is a factor that can be modified via training. Therefore, it can be utilized to for practical assistance in the management of burnout among nurses [14].

The purpose of this study was to investigate the mediating effect of psychological capital on the relationship between job stress and burnout. Demonstrating the mediating effect of psychological capital on the relationship between job stress and burnout among psychiatric nurses. Our findings can be used as a basis for interventional studies that focus on increasing psychological capital in order to reduce job stress and burnout among psychiatric nurses. This will fundamentally reduce job stress and burnout among psychiatric nurses, thereby reducing the turnover rate and helping to improve individual psychological wellbeing and the quality of nursing care.

## 2. Materials and Methods

### 2.1. Study Design and Sample

This study was a descriptive cross-sectional quantitative design using a self-reported survey including a Likert-scale questionnaire.

The subjects eligible for this study were the registered nurses who care for patients in mental health settings. Specific inclusion criteria were as follows: (a) nurses who were currently working in the department of psychiatry, (b) nurses who had worked in the psychiatric unit for more than one year, and (c) nurses who understood the purpose of the research and voluntarily agreed to participate in the research study. The criteria for exclusion were based on prior studies [15,16] with less than one year of mental health work experience. Since experience as a psychiatric nurse was an important criterion, exclusion criteria were set with minimal experience to understand the characteristics of mental health nursing.

The G*Power 3.1.9.4 program was utilized to estimate the sample size of our study. A minimum sample size of 107 was required to obtain a medium effect size (f^2^ = 0.15) with two independent variables and one dependent variable for regression analysis, at a two-sided significance threshold of 0.05 and a power (1 − β) of 0.95; a medium effect size was selected based on previous research to evaluate the mediating effects on the internal characteristics among nurses in the psychiatric unit [17]. Based on the 10% dropout rate suggested in the previous study [18], the final sample size was calculated as 120 questionnaires.

### 2.2. Procedure for Data Collection

Data were collected from 10 August to 15 September 2018. The study participants were convenience sampled from 120 psychiatric nurses were selected. A researcher visited a psychiatric unit of three psychiatry hospitals in South Korea and explained to a nurse manager about permission of the nursing department for the nurse survey, the purpose of the study, and data collection methods. The researcher then asked for their cooperation in order to perform the survey of nurses in their unit. The researcher gave a nurse manager a number of survey questionnaires corresponding a number of nurses in the unit. A total of 120 questionnaires were distributed by hand to the psychiatric nurses through their unit managers. Nurses were asked to put their completed questionnaires without his or her name on it in a sealed box in a designated place in their unit. A researcher took them. About 108 questionnaires were returned, providing a 90.0% response rate. Thus, a total of 108 questionnaires were included in the final analysis.

### 2.3. Instruments

We obtained permission from the original author and translator via email prior to using the study instruments. Three self-reported instruments (job stress, psychological capital, and burnout) used to gather the data.

- Job stress generally means experiencing anxiety, conflict, or pressure in work settings, and it occurs when environmental or internal needs are not met [18]. However, the job stress of psychiatric nurses should include special conditions in the ward, including conflicts in relation with patients who engage in risky behavior, such as suicide or violence, and tension in the unit environment. Therefore, in this study, job stress of psychiatry nurses was measured using the 35 items of the Job Stress Scale, which was modified by Kim, Nam, Lee, and Jeon [19]. Each item was measured using a four-point Likert scoring scale (1 “not at all” to 4 “always”). The total score ranges from 35 to 140, with higher scores indicating higher job stress. Cronbach‘s α was 0.95.
- Psychological capital is a composite concept of self-efficacy, hope, optimism, and resilience which indicates the degree of positive cognitive status of an individual [20]. Psychological capital was measured using the 24 items of the Psychological Capital Scale (Korean version), which was modified by Ko, Park, and Lee [21]. The tool was originally developed by Luthans et al. [20]. Each item was measured using a five-point Likert scoring scale (1 “not at all” to 5 “always”). The total score ranged from 24 to 120, indicating that the higher the score, the higher the psychological capital. Cronbach’s ⍺ was 0.95.
- Brunout originally developed by Pines, Aronson, and Kafry [22] was used. In particular, it was designed to measure the exhaustion of health professionals, such as staff who work at a mental health unit. It was also a tool developed to measure exhaustion at an existential level. This tool defines burnout as being under pressure in some circumstances and unable to cope with stress, and can lead to physical, emotional and mental fatigue and exhaustion. It was measured using the 20 items of the Burnout Scale, which was modified by Moon and Han [23]. Each item was measured using a five-point Likert scoring scale (1 “not at all” to 5 “always”). The total score ranged from 20 to 100, with higher scores indicating higher burnout. Cronbach’s ⍺ was 0.92.

### 2.4. Statistical Analysis

The collected data were analyzed using the IBM SPSS 24.0 software (SPSS Inc., Chicago, IL, USA). First, frequency analysis was performed to identify the demographic characteristics and background of those studied. Second, the t-test and one-way ANOVA were used to examine the differences in burnout by the demographic and careers characteristics. Third, descriptive statistics analysis was conducted to identify the mean and standard deviation of research variables and to identify skewness and kurtosis. Fourth, Pearson’s correlation coefficient analysis was conducted to assess the relationship between study variables. Fifth, Baron and Kenny [24] conducted a multiple linear regression to determine whether psychological capital served as a mediator between job stress and burnout. Additionally, for the control of confounders, we included covariates (confounding variables such as age, clinical career, psychiatric career, violence experience) at one time. To test mediation, we estimated the three following regression equations: first, regressing the mediator (psychological capital) on the independent variable (job stress); second, regressing the dependent variable (burnout) on the independent variable (job stress); and third, regressing the dependent variable (burnout) on both the independent variable (job stress) and on the mediator (psychological capital). Finally, the statistical significance of the mediating effect was verified using a bootstrapping method with the SPSS PROCESS macro (model 4, version 3.4, written by Andrew F. Hayes) [25]. At present, the preferred approach for testing indirect effects is to make use of bootstrapped confidence intervals. Another way to verify the indirect effects is by the Sobel test. However, the Sobel test is overly conservative, with relatively low power and Type I error rates well below the nominal and normative α = 0.05, and users of mediation are advised to avoiding using it [26]. For these reasons, we used a bootstrapping method with higher statistical power and more accurate Type I error rates than Sobel test, even in small samples. Bootstrapping as used in mediation simply resamples the data naively with replacement some number of times (e.g., 5000), and for each sample, estimates of the indirect effect are obtained. Specifically, we calculated a 95% confidence interval (CI) with 5000 bootstrap resamples to determine if psychological capital helped to explain the association between job stress and burnout of psychiatric nurses. After calculation, if a zero does not fall within a 95% CI, there is a 95% likelihood that the indirect effect is significant.

### 2.5. Ethics and Informed Consent

This study was approved by the Institutional Review Board at Chonnam National University (IRB no. 1040198-180618-HR-058-04). Each nursing department of three hospitals permitted survey for their nurses. Participation in this study was voluntary. Nurses were also free to withdraw from the study at any time. When the subjects filled in a survey questionnaire, it was considered as agreement on informed consent. Participants’ anonymity and confidentiality were assured.

## 3. Results

### 3.1. Participant Characteristics

The demographics and careers characteristics of the study subjects are shown in Table 1. The subjects were 90.7% females and 9.3% males. The average age was 38.60 years, with the highest frequency being in their 30s (30.6%), followed by 40s (29.6%), 20s (23.1%), and 50s and older (16.7%). Nurses who were married and those who were divorcees accounted for 62.0% of the subjects, and unmarried women accounted for 38.0%. Most of the subjects had completed undergraduate school (85.2%), while a smaller percentage had completed graduate school (14.8%). The nursing work experience showed that the average clinical experience was 13.39 years, with less than 5 years or more than 20 years making up 25.9% of the subjects each, which accounted for more than half of the study population. These numbers were followed by 5–10 years and 10–20 years at 24.1% each. Almost half of the subjects had psychiatric work experience of less than 5 years (47.2%), followed by over 20 years (21.3%), 10–20 years (17.6%), and 5–10 years (13.9%). About 65.7% of the respondents did not have any turnover experience while working as a nurse, 21.3% of them had once, and 13.0% of them had more than twice. About 95.3% of the respondents had experience of being assaulted.

### 3.2. Descriptive Statistics of Major Variables

The descriptive statistics on research variables are shown in Table 2. The average score of job stress was 88.03 points. The average score of psychological capital was 85.54 points, and the average score of burnout was 53.79 points. All variables used in this research did not exceed an absolute value of skewness of 3.0 and an absolute value of kurtosis of 10.0, thus satisfying univariate normality.

### 3.3. Correlations between Major Variables

The relationships between major variables are shown in Table 3. Burnout of psychiatric nurses showed a significant correlation with all variables. Specifically, burnout was positively correlated with job stress (r = 0.55, *p* < 0.001), but negatively correlated with psychological capital (r = −0.65, *p* < 0.001). Furthermore, job stress was negatively correlated with psychological capital (r = −0.48, *p* < 0.001).

### 3.4. Mediating Effects of Psychological Capital on the Relationship between Job Stress and Burnout

The results of the mediating effects of psychological capital on the job stress and burnout relationship among psychiatric nurses are shown in Table 4. In this study, the three-step method [23] was used to analyze the mediating effect. In the first step, we analyzed the effect of job stress on psychological capital as a mediating variable. This confirmed that job stress had a significant effect on psychological capital (β = −0.48, *p* < 0.001). In step two, we analyzed the effect of job stress on burnout as a dependent variable. As a result, job stress had a significant effect on burnout (β = 0.55, *p* < 0.001). In the third step, psychological capital, which is a parameter, was added and input as an independent variable along with job stress, and burnout was added as a dependent variable. As a result, job stress had a statistically significant effect on burnout (β = 0.30, *p* < 0.001) and also psychological capital had a significant effect on burnout (β = −0.51, *p* < 0.001). These results suggest that job stress and burnout are partially mediated by psychological capital. Job stress explained 29.7% of the variance in burnout, and the model including job stress and psychological capital explained 49.6% of the variance in burnout.

We conducted bootstrapping using the PROCESS macro to verify the mediating effects. Table 5 shows the direct and indirect effects of the mediating variable. There were 5000 samples re-extracted from bootstrapping, and the lower and upper limits of the indirect effect coefficient were analyzed in the 95% confidence interval. The analysis showed that the indirect effect was 0.2417, which was strong enough to account for 58.8% of the total effect. As a result of the mediating effect test, psychological capital between job stress and burnout did not include zero between the lower limit value (0.1442) and the upper limit value (0.3548) of the confidence level. Therefore, this was statistically significant.

## 4. Discussion

Psychiatric nurses have a key role in taking care of patients with mental health problem, being in many difficult situations. They are also likely to be exhausted because they have been in a dangerous and stressful situation for a long time. Therefore, it is important to prevent their job stress and burnout. This study attempted to explore the role of psychological capital between job stress and burnout of psychiatric nurses, seeking strategies to reduce their burnout. Discussions based on research results are as follows.

First of all, this study sought to identify the levels of job stress and burnout among Korean psychiatric nurses. The results show high levels of job stress and burnout of psychiatric nurses in South Korea. The score of job stress was 88.03 ± 17.45, and the findings are similar to those of the previous studies on psychiatric nurses [4,27]. Another previously study in Saudi Arabia produced results that are in line with the results of this study [28]. These studies showed that psychiatric nurses have a higher job stress than nurses who work elsewhere in non-psychiatric settings. The nurse’s job stress has a direct and negative effect on the patient. In particular, evidence suggests that nurses’ job stress affects patient safety [27,28,29,30]. Furthermore, the primary task of psychiatric nurses is to communicate with patients who have mental health difficulties and their families. They should also provide guidance on how to deal with the psychiatric problems that patients are experiencing. Therefore, the psychiatric nurse’s job stress must be managed and prevented.

In this study, the burnout score was 53.79 ± 13.16 on average. In addition, the factors affecting burnout were age, clinical career, career at the department of psychiatry, experience being assaulted. Those who were younger, had more experience of being assaulted, less working experience as a nurse, and less psychiatric career experience showed a higher burnout score. The results reveal that there were many factors that contributed to burnout. These results are consistent with the previous studies [31,32,33] on influencing factors and burnout among psychiatric nurses. The previous studies of psychiatric nurses showed high burnout, similar to those of this study [34,35,36]. Burnout among nurses can lead to them suffering from fatigue, insomnia, headaches, anger, and depression [37]. There may be differences depending on the unique environment of psychiatry [38]. For instance, a significant relationship was found between workplace and communication subscale in mental health setting. Additionally, psychiatric nurses constantly manage patients and families from all backgrounds, and it is essential to deal with manifold communications. Both injuries from work and verbal attacks from patients will increase psychiatric nurses’ burnout. When psychiatric nurses are hurt at work and verbally attacked by patients, the burnout level is high [39]. Similarly to this study, Hamaideh [40] made a suggestion that drew attention to the importance of improving the psychosocial work environment among mental health nurses, thus affecting the burnout. For instance, to decrease burnout among psychiatric nurses, administrators should provide education to deal with the aggressive behavior of patients, as well as programs to deal with and manage stress so that younger or less-experienced nurses are not so easily burned out.

Second, this study aimed to investigate the relationships between job stress, psychological capital, and burnout among psychiatric nurses. In this study, the results reveal that there were strong relationships among the variables. Job stress among psychiatric nurses showed a significant correlation with burnout. Our findings indicate that psychiatric nurses who are stressed due to their work are significantly more likely to experience burnout. This result supports the previous studies, which showed that nurses who get stressed tend to experience physical and mental health issues [27,28]. Psychiatric nurses have higher job stress levels compared to nurses from non-psychiatric settings because psychiatric nurses experience pressure while dealing with many psychiatric patients in-person [3,12,29]. Moreover, psychiatric nurses experience job stress due to tension and strain, as they encounter dangerous situations (including harmful behavior for themselves or other patients and acute symptoms) sporadically and are faced with a shortage of manpower [3,38]. These working situations in psychiatric wards can lead to many harmful outcomes, such as stress, tension, and dissatisfaction [39,40,41]. From a workplace safety-related perspective, it is very important to manage the job stress of nurses working in psychiatric wards. Based on the fact that nurses’ burnout results from long-term stress in the workplace, it is important to take care of the job stress of psychiatric nurses in the clinical field. Moreover, when the job stress is excessive, it can cause a negative attitude among psychiatric nurses. Therefore, it is necessary to make an effort to change internal factors in order to handle the inevitable stress.

Psychological capital of psychiatric nurses was found to have a negative correlation with job stress and burnout in this study. Job stress showed a negative correlation with psychological capital, and it can be predicted that psychological capital will decrease when job stress increases. Consequently, the higher the psychological capital of the psychiatric nurse, the lower the job stress and burnout. This result is similar to the previous study [42], which found that the psychological capital of clinical nurses lowers the rate of burnout, and the rate of burnout decreases as the positive influence of the individual increases. In other words, the higher the psychiatric nurse’s psychological capital, the lower his or her rate of burnout. From this point of view, we should help to prevent the burnout of psychiatric nurses by increasing psychological capital. Furthermore, in terms of organization management, the burnout of psychiatric nurses should be managed. Burnout of psychiatric nurses can lead to negative consequences in their relationship with patients, and reduced quality of services provided to patients and clients [6,7,43]. Additionally, nurses’ burnout can lead to decreased work efficiency, increased absenteeism, and personnel turnover [43,44,45]. Therefore, it is very important to prevent the burnout of psychiatric nurses by managing the job stress and psychological capital associated with the burnout of psychiatric nurses.

Finally, the main contribution of this study is to highlight that job stress may affect the risk of burnout in psychiatric nurses via a mediating mechanism of psychological capital. Our study results identified psychological capital as a positive resource for reducing the negative effects of job stress on burnout. In other words, it was determined that psychological capital partially mediated the relationship between job stress and burnout. Furthermore, this study’s results suggest that job stress explained 29.7% of the variance in burnout, and the model including job stress and psychological capital explained 49.6% of the variance in burnout. We found that job stress among psychiatric nurses directly affects burnout, and psychological capital indirectly affects burnout. Job stress level was a unique predictor of burnout among health care facilitators. There is no doubt that job stress and burnout are closely related. Most studies have revealed that high levels of job stress increase the level of burnout of nurses [46]. Meanwhile, psychological capital can be seen as an important human resource that has significant effects on job stress and the burnout of psychiatric nurses. Luthans and Jensen [41] believed that psychological capital may effectively reduce the extent of burnout. A previous study in China found preliminary evidence for the preventative effect of psychological capital on burnout [47]. Other studies have also suggested that increasing psychological capital is important to prevent and relieve the burnout of nurses within limited resources, along with decreasing job stress [21,48]. Our study is meaningful because it identifies that psychological capital, which was found to make self-development possible and affect individuals and organizations positively in previous studies [49], is a parameter of job stress and burnout. Above all, the psychological capital, or the parameter in this study, will have a positive effect on psychiatric nurses who are physically and mentally weak and exhausted. Moreover, it will contribute to the improvement of the quality of nursing care and the human resource management of nursing personnel. Our research findings emphasize the importance of developing psychiatric nurses’ psychological capital in fostering their quality of care.

The limit of our research is that the research design is a cross-sectional design. This research design allows relationships between variables to be identified at one point of time only and does not allow causal relationships among variables to be established. Therefore, to better understand the burnout of psychiatric nurses, longitudinal designs are recommended for future studies. Additionally, this study only investigated the impact of psychological capital on the relationship between job stress and burnout of psychiatric nurses. Factors other than psychological capital may also have similar influences, such as self-esteem, social support, and working environment. It is suggested that future research be carried out by involving other factors related to the burnout of nurses working in the psychiatric ward. In addition, the data excluded from the analysis in this study comprised 10% of all subjects. Since missing data may affect the results of the study, it is important to be careful of the magnification.

## 5. Conclusions

This study was conducted to examine the relationship between job stress, burnout, and psychological capital among psychiatric nurses, to analyze the mediating effect of psychological capital on the relationship between job stress and burnout, and to present data for developing strategies in efforts to reduce burnout of psychiatric nurses.

Our results show that psychological capital among psychiatric nurses was identified as a major predictor of burnout, and it also had a partial mediating effect on job stress and burnout among psychiatric nurses. Therefore, in order to reduce the burnout of psychiatric nurses, this study suggests improving the psychological capital of psychiatric nurses by developing and implementing mediating programs.

## Figures and Tables

**Table 1:** Participant characteristics, differences of burnout by characteristics.

Characteristics	Categories	n (%)	M ± SD	Burnout	
				M ± SD	t/F
Gender	Male	10 (9.3)		60.70 ± 12.00	1.76
	Female	98 (90.7)		53.08 ± 13.12	
Age (yrs.)			38.60 ± 9.39		
	20~29	25 (23.1)		58.08 ± 14.53	2.87 *
	30~39	33 (30.6)		55.34 ± 10.82	
	40~49	32 (29.6)		52.34 ± 12.80	
	≥50	18 (16.7)		47.17 ± 14.44	
Marital status	Married	41 (38.0)		55.73 ± 14.08	1.21
	Unmarried	67 (62.0)		52.52 ± 12.66	
Education status	Undergraduate	92 (85.2)		53.29 ± 13.12	1.93
	Graduate	16 (14.8)		50.31 ± 9.73	
Clinical career (yrs.)			13.39 ± 9.71		
	<5	28 (25.9)		55.32 ± 13.40	3.26 *
	5~10	26 (24.1)		57.88 ± 13.28	
	10~20	26 (24.1)		54.69 ± 11.22	
	≥20	28 (25.9)		47.61 ± 12.94	
Psychiatric careers(yrs.)			10.67 ± 9.90		
	<5	51 (47.2)		56.04 ± 130.4	2.80 *
	5~10	15 (13.9)		54.60 ± 11.79	
	10~20	19 (17.6)		55.37 ± 11.40	
	≥20	23 (21.3)		46.96 ± 14.05	
Number of turnover	None	71 (65.7)		53.49 ± 13.15	0.18
	1 time	23 (21.3)		55.43 ± 12.86	
	≥2 times	14 (13.0)		52.42 ± 15.50	
Violence experience	None	5 (4.7)		51.20 ± 7.59	3.77 *
	Sometimes	79 (73.1)		52.03 ± 12.81	
	Often	24 (22.2)		60.13 ± 13.47	

Note: M = mean; SD = standard deviation; t = values of t-test; F = values of ANOVA; * *p* < 0.05; yrs = years.

**Table 2:** Descriptive statistics of research variables.

Variables	Range	M ± SD	Skewness	Kurtosis
Job stress	35~140	88.03 ± 17.45	−0.279	−0.315
Psychological capital	24~120	85.54 ± 14.33	−0.433	−0.168
Burnout	20~100	53.79 ± 13.16	0.048	−0.607

Note: M = mean; SD = standard deviation.

**Table 3:** Correlations between research variables.

Variables	Job Stress	Psychological Capital	Burnout
Job stress	1		
Psychological Capital	−0.48 ***	1	
Burnout	0.55 ***	−0.65 ***	1

Note: *** *p* < 0.001.

**Table 4:** Mediating effects of psychological capital on the relationship between job stress and burnout.

Step	Pathway	B	SE	β	t	Adj.R^2^	F
1	Job stress→Psychological capital	−0.39	0.070	−0.48	−5.58 ***	0.227	31.13 ***
2	Job stress→Burnout	0.41	0.061	0.55	6.68 ***	0.297	44.74 ***
3	Job stress→Burnout	0.22	0.059	0.30	3.84 ***	0.496	51.59 ***
	Psychological capital→Burnout	−0.46	0.072	−0.51	−6.43 ***		

Note: B = unstandardized regression coefficient; SE = standard error; β = standardized regression coefficient; t = values of t-test; Adj. R^2^ = Adjusted coefficient of determination; F = values of ANOVA; *** *p* < 0.001.

**Table 5:** Verifying the bootstrapping mediation effect.

Psychological Capital	B	SE	Bootstrap 95% CI
Indirect effect	0.2417	0.0540	[0.1442, 0.3548]
Direct effect	0.2286	0.0594	[0.1107, 0.3465]
Total effect	0.4108	0.0614	[0.2891, 0.5326]

Note: B = unstandardized regression coefficient; SE = standard error; CI = confidence interval.
